# Mindfulness-Based Cognitive Therapy as Migraine Intervention: a Randomized Waitlist Controlled Trial

**DOI:** 10.1007/s12529-021-10044-8

**Published:** 2021-12-21

**Authors:** K. Simshäuser, R. Pohl, P. Behrens, C. Schultz, C. Lahmann, S. Schmidt

**Affiliations:** 1 Department of Psychosomatic Medicine and Psychotherapy, Medical Faculty, Medical Center, University of Freiburg, Hauptstraße 8, 79104 Freiburg, Germany; 2 Interdisciplinary Pain Center, Medical Faculty, Medical Center, University of Freiburg, Freiburg, Germany; 3 University Center for Complementary Medicine, Medical Faculty, Medical Center, University of Freiburg, Freiburg, Germany; 4 Institute for Frontier Areas of Psychology and Mental Health, Freiburg, Germany

**Keywords:** Migraine, Mindfulness, CBT, MBCT, RCT

## Abstract

**Background:**

Based on promising effects seen in a pilot study evaluating a generic mindfulness-based program for migraine, we developed a migraine-specific adaptation of the Mindfulness-Based Cognitive Therapy (MBCT) program. The aim of this study was to evaluate this program for feasibility and effectiveness in a randomized controlled trial.

**Method:**

Fifty-four patients suffering from migraine were randomly allocated to either waitlist or the adapted MBCT. Outcomes were migraine-related parameters as well as variables of psychological functioning and coping. Assessment took place at baseline and post-intervention, for the intervention group also at follow-up (7 months). The effects of the intervention were analyzed by the use of ANCOVAs and linear mixed models.

**Results:**

With respect to migraine parameters we did not find a significant group difference in the primary outcome (headache-related impairment), but the intervention resulted in a significant reduction of headache frequency (*p* = .04). In the analysis of secondary outcomes, MBCT showed superiority in four out of eight psychological parameters (perceived stress, anxiety, rumination, catastrophizing) with small to medium effect sizes. The intervention proved to be feasible and participants reported high degrees of contentment and achievement of personal goals.

**Conclusions:**

The migraine-specific MBCT program did not result in improvements with regard to headache-related impairment but showed a reduction in headache frequency as well as improved psychological functioning in secondary outcomes.

**Trial Registration:**

This trial was registered in the German Trial Registry “Deutsches Register Klinischer Studien” (ID: DRKS00007477), which is a WHO-listed primary trial register.

**Supplementary Information:**

The online version contains supplementary material available at 10.1007/s12529-021-10044-8.

## Introduction

Migraine is a widespread condition and affects about 12% of the overall population [1]. Its economic burden is substantial [2–4] and the majority of people suffering from migraine report reduced quality of life and considerable functional impairment at work and in family life [5–8]. Also, in the pain-free interictal intervals between attacks, migraine patients report heightened levels of stress reactivity and emotional irritability [9] in their everyday life, as well as worries and anticipatory fear about the next attack [10].

Apart from pharmacological treatment, psychological interventions are recommended as a prophylactic treatment. Here, cognitive-behavioral therapy (CBT) is seen as a promising approach [11, 12], but it is limited, showing moderate effect sizes and not being relevant for a substantial amount of patients [13, 14]. Likewise, mindfulness-based programs like Mindfulness-Based Stress Reduction (MBSR) appear as new treatment options for migraine. Recent meta-analyses assessing these interventions in the field of (chronic) primary headache revealed a wide range of methodological quality across the assessed randomized controlled trials (RCTs) with inconclusive results up to this point, especially concerning their effects on direct headache parameters [15, 16].

As an innovative approach, Day and Thorn [17–19] introduced *Mindfulness*-*Based Cognitive Therapy* (MBCT) into headache treatment, complementing CBT with principles of mindfulness. While CBT emphasizes and modifies the connection between cognition, emotion, and behavioral patterns, MBCT aims at shifting the person’s relationship to these phenomena at a meta-cognitive level. Through mindfulness meditation, a decentered perspective of observing one’s thoughts and sensations without judging is trained, fostering acceptance towards experiences including pain sensations [17]. In this way, a change-based CBT approach of recognizing and modifying thought and behavior patterns is combined with a more acceptance-based mindfulness approach of developing a less reactive and less identified “observer position” towards (painful) experiences.

Since MBCT shows promising results in depression relapse prevention [20] and anxiety treatment [21] so far, Day and Thorn adapted the original MBCT program from Segal et al. [22] to headache disorders. They introduced their adapted program as a new treatment option for headache patients. In a RCT with 36 patients with primary headache, the authors found significant improvements in psychological variables like pain acceptance and pain self-efficacy compared to a waitlist control [23]. Headache-related outcomes such as frequency, intensity, and disability showed improvements in the predicted direction despite limited study power. However, in a per-protocol analysis the outcome headache-related disability showed a significant superiority compared to the control condition with a large effect size (*d* = 1.29).

In a similar way, Seng et al. [24] recently adapted the MBCT manual to migraine independently from our own research. The authors evaluated a MBCT program in 60 migraine patients in a RCT and compared it to a waitlist condition. Notably, the MBCT program was delivered on a one-to-one basis. The authors found a significant superiority in two different measures of headache-related disability, but no effect on migraine frequency. They concluded that the adapted MBCT is a promising treatment option in order to address migraine-related disability.

A slightly different pattern of results was shown by Wells et al. [25] using the generic (not migraine adapted) MBSR intervention. The authors evaluated MBSR in a sample of 89 migraine patients against an active control group. Unlike in the study of Seng et al. [24], MBSR reduced migraine frequency significantly, although not better than the active control. Similar to the study of Seng and colleagues, MBSR was found to be superior to the control group in headache-related disability and in variables of psychological functioning with medium to large effects, affecting the overall burden of disease according to the authors’ conclusion. A recent trial by Seminowicz et al. [26] also compared an enhanced and extended MBSR program (12 sessions within 4 months) to an active control group and found significant effects for number of headache days and headache-related impairment.

In our own pilot RCT, we also evaluated a generic Mindfulness-Based Stress Reduction (MBSR) program in 62 people with migraine compared to progressive muscle relaxation [27]. MBSR was not found to be superior in most parameters, as the pilot study was primarily conducted to estimate feasibility and lacked statistical power due to loss of patients in the control condition. However, headache-related disability decreased in the MBSR condition showing a medium effect size, influencing the choice of the primary outcome in our current study. In order to further enhance the effectiveness of the mindfulness-based approach, we chose the headache-specific MBCT program developed by Day and Thorn and adapted it to migraine with the kind permission of the authors.

Finally, the objective of our study was to evaluate the feasibility and effectiveness of a migraine-adapted, group-based MBCT program in a RCT. We hypothesized that the program would be a safe and feasible intervention for moderately impaired migraine patients. Furthermore, we hypothesized that patients treated with MBCT would show significant improvements in migraine parameters and variables of psychological functioning and coping compared to a waitlist control group.

## Methods

### Design

We conducted a randomized controlled trial with a waitlist control group. Measurements were taken at the beginning of the intervention (*t*0), at the end of the intervention (*t*1) and for the intervention group after a 7-month follow-up (*t*2).

### Participants

Participants were recruited via local advertisements, local neurologists and the Pain Unit of the Medical Center of the University of Freiburg. Inclusion criteria were as follows: (1) aged 18–65 years, (2) diagnosis of migraine with or without aura by the trial physician in accordance with the diagnostic criteria of the International Headache Society [28], (3) at least two migraine attacks per month on average, and (4) in case of a medical prophylaxis maintaining a stable dose for at least 3 months prior to inclusion until the end of the trial. Exclusion criteria were as follows: (1) chronic migraine with more than 15 migraine days per month, (2) taking headache analgesics on more than 15 days or migraine-specific triptans on more than 10 days per month, (3) regular practice of meditation (> 1 × per week) or yoga (> 2 × per week), (4) plans to start psychotherapy or any other migraine treatments during the course of the trial, (5) prior participation in a mindfulness training, (6) participation in other clinical studies throughout the study duration, and (7) presence of a life-threatening disease or a mental disorder that might severely hinder interpersonal contacts. Taking acute headache medications like non-steroidal anti-rheumatics (NSAR) and triptans during the trial period was allowed.

### Procedure

The patients were recruited between Nov 2014 and Feb 2015. They underwent initial telephone screening and, if potentially eligible, were invited to the study center at the Medical Center of the University of Freiburg for examination by one of three study physicians and the study psychologist. They were provided with information about the trial and gave written informed consent. Next, the participants were asked to complete a first set of questionnaires and received a headache diary with detailed instructions to be filled in daily for the next 4 weeks (*t*0). The patients were allocated to one of the two trial arms and were informed in writing about the result of the allocation in the middle of their diary assessment. The patients allocated to the MBCT arm started their course a few weeks later and completed their post-assessment (*t*1) headache diaries and questionnaires shortly afterwards. Headache diaries and questionnaires were provided in the last MBCT session and were sent back by mail by the participants 4 weeks later. The patients allocated to the waitlist group did not receive any treatment within that period, but were sent the same measurements at the same time (*t*1) by mail. They received the MBCT intervention after their post-assessment. A 7-month follow-up (*t*2) completed the assessment for the MBCT group; the participants were sent diaries and questionnaires by mail and were asked to send them back. Based on the waitlist design, the participants could not be blinded with respect to their group allocation. Data entry was performed in a blinded manner. Data analysis was not performed in a blinded manner, as the principal investigator of the study was responsible for patient recruitment, patient guidance, data collection, and data analysis.

### Randomization

The patients were assigned to groups through the design-adaptive allocation approach by Aickin [29–31]. This regression-based minimization procedure takes advantage of creating balanced groups of participants with respect to chosen prognostic variables. We used age, gender, and self-reported (baseline) number of migraine days per month as factors. An anonymous list of included patients was sent to Mikel Aickin in Arizona. He performed the group allocation according to this method and generated and returned two balanced lists of participants. Finally, a person not further involved in the trial randomly allocated the group assignment of the two lists.

### The Migraine-Adapted MBCT Intervention

The formal structure of our adapted MBCT group intervention closely followed the manual by Day [17], which in turn closely parallels the MBCT manual for depression relapse from Segal et al. [22]. The intervention consisted of eight weekly 2.5-h sessions. At the start an individual intake interview was held with the MBCT teacher in order to assess personal goals and motivations. Finally, a booster session for refreshment was held after 6 months. Overall, four courses (two courses for the invention group and two delayed courses for the control group) were conducted with an average group size of 12 participants. The courses were held by three experienced and certified MBSR/MBCT teachers from the local mindfulness network. In order to ensure expertise with the disease, the three teachers were provided with a professional education by one of the study physicians. In order to ensure adherence to the MBCT protocol, the principal investigator of the study and the teachers met regularly for discussions during the study interval. In these sessions, the specific adaptations of the manual were discussed in detail.

Regarding program content, the depression-related cognitive-behavioral elements of the original MBCT from Segal et al. [22] were transformed to headache-specific adaptations by Day and Thorn [32], which were then further adapted to migraine-specific adaptations by our research group. This encompassed (1) educational elements about the condition of migraine, (2) fostering self-monitoring of the cascades of thoughts, feelings, and bodily reactions, (3) identifying cognitive errors, (4) regulating the level of activity and stress in everyday life, and (5) fostering early recognition and regulation of specific signs of stress and overload. The participants were encouraged to practice at home for 30–45 min a day.

### Outcomes

Outcomes were chosen with regard to the recommendations of the IMMPACT (Initiative on Methods, Measurement, and Pain Assessment in Clinical Trials) group [33]. A headache diary assessed headache-related impairment as well as migraine-specific related outcomes. Psychological variables of functioning and coping were assessed via self-report questionnaires. Socio-demographic and medical history features as well as treatment satisfaction, compliance, and degree of goal achievement were assessed with the aid of self-constructed questionnaires.

#### Primary Outcome

Primary outcome was the group difference at *t*1 of the variable “headache-related impairment.” It was assessed via three items asking for impairment in everyday life, at work and during leisure. Items were assessed on an 11-point numeric rating scale (0–10) with the anchors “no interference at all” to the “most severe interference”. These items were taken from the Pain Disability Index [34, 35], and they are also used in the German Pain Questionnaire [36]. They were assessed on a daily basis in a headache diary (see below). Cronbach’s *α* for the three items in our sample at baseline (*t*0) was 0.93 (0.91 at *t*1; 0.93 at *t*2). Proof of validity has been provided by the authors, including correlations with the degree of behavioral disability in the everyday life, the amount of time spent in bed due to pain or the level of depression. The authors state that the three items can be aggregated to an overall value of pain-related disability. We calculated an overall disability value for every headache day. We then aggregated these values over 1 month to a monthly disability value, which served as our primary outcome.

#### Secondary Outcomes

#### Migraine-Related Outcomes

The participants were provided with headache diaries to be completed daily for 4 weeks at pre- and post-intervention for both groups, and also at 7-month follow-up for the intervention group. It consisted of eight columns assessing headache intensity, headache-related impairment, headache characteristics (duration, pain character, aggravation by exercising, presence of the three attendant symptoms aura, sensitivity to light and noise, nausea and sickness), and medication use. Headache intensity was assessed on a numeric rating scale (0–10) ranging from “no pain at all” to the “most severe pain.” In case of a day with no headache, the patients marked the first column (headache intensity) with an “X” and did not fill in the other columns. In case of a headache day, all eight columns had to be filled in. The patients were instructed to fill in this first column in any case, in order to retrieve the number of headache days per month without any missings or need for imputation. Headache characteristics were assessed with dichotomous items (i.e., a duration more or less than 4 h). For assessing medication use, the exact acute medication should be noted for each day. Medication use is reported as number of days with acute medication per month. Headache frequency reflected the number of days per month with any kind of headache. Both variables were converted to a standard month of 30 days.

#### Psychological Outcomes

The participants filled out a battery of the following questionnaires at all three measurement points. All the standardized questionnaires showed at least satisfactory reliability and validity in clinical trials as well as suitability in pain patient samples.

##### Hospital Anxiety and Depression Scale

To assess the degree of affective disturbance, the Hospital Anxiety and Depression Scale (HADS-D) was used [37–39]. The HADS-D is a well-established, low-threshold screening instrument for assessing anxious and depressive symptoms in patients with somatic complaints. We found in our sample a Cronbach’s *α* for anxiety of 0.78 (*t*0), 0.83 (*t*1), and 0.88 (*t*2), and for depression of 0.79 (*t*0), 0.82 (*t*1), and 0.88 (*t*2) respectively.

##### Perceived Stress Questionnaire

The degree of stress in everyday life was assessed with the Perceived Stress Questionnaire (PSQ) [40, 41]. The PSQ measures the amount of subjectively experienced stress, according to inner experiences like having sorrows, feeling tension, experiencing lack of positive emotions, and being confronted with requirements. Cronbach’s *α* was 0.91 (*t*0), 0.93 (*t*1), and 0.96 (*t*2) in our sample.

##### Perceived Stress Reactivity Scale

The level of stress reactivity was assessed via the Perceived Stress Reactivity Scale (PSRS) [42, 43]. The PSRS asks for the duration and the extent of a person’s affective reaction in stressful situations as a dispositional trait. We found in our sample a Cronbach’s *α* of 0.89 (*t*0), 0.91 (*t*1), and 0.86 (*t*2).

##### Questionnaire of Dysfunctional and Functional Self-Consciousness

The amount of rumination was assessed through the scale “dysfunctional self-attention” of the Questionnaire of Dysfunctional and Functional Self-Consciousness (DFS) [44]. It assesses to what extent people are directing their attention inflexibly towards their inner processes and are persevering in these when confronted with disturbances or obstacles. Cronbach’s *α* was 0.90 (*t*0), 0.92 (*t*1), and 0.92 (*t*2).

##### Pain-Related Self Statements Scale

The amount of catastrophic thinking in reaction to pain is assessed with the scale “catastrophizing” of the Pain-Related Self Statements Scale (PRSS) [45, 46]. Statements that indicate higher levels of this mode of thinking reflect higher states of helplessness and hopelessness in the face of pain. Cronbach’s *α* was 0.84 (*t*0), 0.87 (*t*1), and 0.78 (*t*2) in our sample.

##### Self-Compassion Scale

The Self-Compassion Scale (SCS) assesses the degree of self-directed compassion or empathy which expresses a positive attitude of a person towards him/herself even in the face of inadequacies, failures or difficult life circumstances [47, 48]. The overall value of this questionnaire has shown a Cronbach’s *α* of 0.91 (*t*0), 0.94 (*t*1), and 0.95 (*t*2) in our sample.

##### Freiburg Mindfulness Inventory

The short-version of the Freiburg Mindfulness Inventory (FMI) targets the amount of self-reported mindfulness by assessing the underlying non-judging, accepting, and non-identifying qualities of the mindfulness construct [49, 50]. We found a Cronbach’s *α* of 0.78 (*t*0) 0.88 (*t*1), and 0.82 (*t*2) in our sample at baseline.

#### Socio-demographic Data, Feasibility and Goal Attainment Assessment

Additionally, we administered questionnaires assessing personal data on socio-demographic features and characteristics of the migraine disease at baseline. For the feasibility assessment, we assessed treatment satisfaction and homework adherence via questionnaires including Likert scales at the follow-up-assessment. As a final measure, two personally relevant goals from every patient were assessed in the interview session with the MBCT teacher with the use of a goal attainment assessment [51]. At the end of the course, these sheets were given to the patients again along with the other questionnaires in order to rate their goal attainment on a five-point scale (from −2 “achieved goals much less than expected” to + 2 “achieved goals much more than expected”).

### Data Analysis and Statistics

#### Statistical Power

Our study was designed to find a medium to large effect on the primary outcome in a waitlist design. In our pilot study, the variable of migraine-related impairment showed an effect size of *d* = 0.66. By specifying our intervention to our patient sample, we sought to increase this effect to at least 0.7. To detect an effect of *d* = 0.7 with a statistical power (1 − *β*) of 0.80, a sample size of *n* = 52 would be needed.

#### Missing Data

For the headache diary, missing data at post- or follow-up assessment were replaced through the last-observation-carried-forward procedure (LOCF). This procedure was done at the level of aggregated mean values for all headache variables (i.e., impairment, intensity, number of headache days, and number of medication days). In the MBCT group, the diaries at baseline from three people were found to provide invalid data due to a large amount of missing data (> 50%). They were not included in the analyses, reducing the data set to *n* = 24. In the control group, one person provided data with a large portion of missings. Two other patients who left the study did not return their diaries, reducing the diary-based data set to *n* = 24. For the clinical questionnaires, missing data were first checked for random distribution and were then replaced with the expectation–maximization algorithm [52]. The critical rate of 30% of missing values (i.e., single fields in the diary) for the application of the algorithm was at no time exceeded [53]. As predictor variables for the expectation–maximization algorithm, baseline values as well as the three stratification variables of the design-adaptive allocation were chosen. Here, all 54 patients provided valid data sets and could be included.

#### Data Analysis

All the analyses were conducted with SPSS Statistics 18, and the statistical software R. Differences between groups from baseline to post-measurement were assessed via analysis of covariance (ANCOVA). Baseline scores as well as stratification variables were entered as covariates in a model with group as the independent factor. As partial Eta^2^ (*η*^2^) as a common measure of effect size in analyses of variance has no pre-defined range, Cohen’s *d* was computed instead. As effect size measure for the ANCOVA, we calculated an adjusted Cohen’s *d* (*d*_adj_) by dividing the difference of the adjusted means through the pooled standard deviation at post-measurement.

Variables with frequency data, i.e., number of headache days and number of medication days, are assumed to be Poisson rather than normally distributed. For them a linear mixed model based on a Poisson distribution with the use of the package lme4 in R was computed. We assessed the *time* × *group* interaction. Additionally, the stratification variables age, gender, and self-reported days of migraine at intake were entered into the model as fixed factors, while intercept was entered as a random factor.

Regarding correction for multiple testing of secondary outcomes, we refer to the recommendations of Schulz and Grimes [54]. The authors state that formal corrections like the Bonferroni correction inflate the type-II-error and devaluate information stemming from trials with many endpoints. We followed their recommended procedure to set one primary endpoint that will be prioritized for the assessment of the trial and to only report results that have been set by the study protocol.

## Results

### Participants

**Fig. 1:**
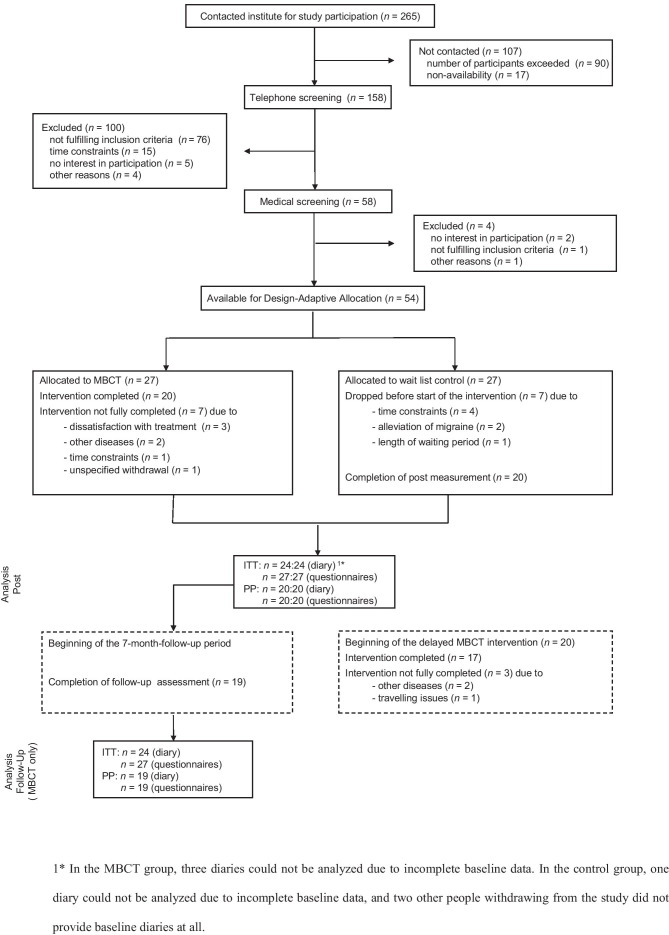
Participants’ flow

The participants’ flow following the CONSORT statement [55] is depicted in Fig. 1. After telephone screening and intake examination, 54 participants were enrolled in the trial. The recruitment process was terminated as soon as the planned number of participants was included. In both groups, seven people dropped out of the trial over the course of the study, resulting in 20 people providing post-data in each group. Of the 20 people completing the MBCT intervention, 19 provided a follow-up assessment 7 months later.

Table 1 provides an overview of the sample’s demographic and clinical characteristics by group assignment. There were no significant differences between the groups in any of the demographic and clinical measures, nor were there differences according to baseline values of all outcome (diary and questionnaire) variables. There were no significant differences between the people who dropped out of the trial and study completers in socio-demographic and clinical values at baseline.

**Table 1:** Baseline characteristics of patients randomized to MBCT and control group as well as *p*-values of *t*-tests (continuous data) or Fisher’s exact test (frequency data)

	**MBCT *n* = 27**	**Control *n* = 27**	***p-value***
**Age**; *M (SD)*	44.4 (8.86)	46.1 (12.11)	.55
**Gender**			
Male (%)	7.4	14.8	.67
Female (%)	92.6	85.2	
**Onset of disease** (%)			
1–5 years ago	3.7	3.7	1.00
5–10 years ago	14.8	11.1	
> 10 years ago	81.5	85.2	
**Days with migraine in the last month**; *M (SD)*	6.04 (3.04)	5.30 (2.61)	.34
**Degree of impairment during an acute migraine attack** (0–10); *M (SD)*	8.26 (1.35)	7.96 (1.72)	.48
**Intake of acute medication (yes-response)** (%)	96.3	100	1.00
**Intake of prophylactic medication** (%)	14.8	7.4	.67

*M* mean, *SD* standard deviation, *p p*-value for Fisher’s exact test / *t*-test. The variables *Age*, *Gender*, and *Days with migraine in the last month* were used as stratification variables

Table 2 provides an overview of the descriptive statistics for all variables in this trial.

**Table 2:** Descriptive statistics for all headache-related and psychological variables

		***t*0**		***t*1**		***t*2**	
		*M*	*(SD)*	*M*	*(SD)*	*M*	*(SD)*
**Impairment during a headache attack (0–10)**	MBCT Control	4.41 4.40	*(2.44)* *(1.56)*	4.05 4.39	*(1.90)* *(1.60)*	4.25 -	*(2.42)* *-*
**Pain intensity during a headache attack (0–10)**	MBCT Control	3.88 4.26	*(1.32)* *(1.26)*	4.24 4.29	*(1.31)* *(1.58)*	4.16 -	*(1.62)* *-*
**Number of headache days per month**	MBCT Control	8.17 8.50	*(4.71)* *(5.59)*	5.96 8.38	*(3.58)* *(7.32)*	5.04 -	*(3.56)* *-*
**Number of days with medication per month**	MBCT Control	4.58 5.54	*(2.54)* *(2.55)*	4.00 4.08	*(2.40)* *(2.39)*	3.46 -	*(2.28)* *-*
**PSQ (0–100)**	MBCT Control	45.41 47.48	*(17.54)* *(15.99)*	38.80 48.20	*(18.55)* *(17.85)*	42.88 -	*(22.50)* *-*
**HADS-D — anxiety (0–21)**	MBCT Control	6.63 6.74	*(3.88)* *(2.90)*	6.02 7.29	*(4.17)* *(3.75)*	7.42 -	*(4.28)* *-*
**HADS-D — depression (0–21)**	MBCT Control	4.22 5.33	*(2.55)* *(3.63)*	3.80 5.19	*(2.93)* *(2.98)*	4.97 -	*(3.78)* *-*
**PSRS (0–46)**	MBCT Control	25.44 28.44	*(8.18)* *(7.77)*	23.22 27.72	*(7.96)* *(8.49)*	24.40 -	*(6.22)* *-*
**DFS — rumination (1–5)**	MBCT Control	3.07 3.32	*(0.66)* *(0.69)*	2.80 3.28	*(0.67)* *(0.72)*	2.87 -	*(0.77)* *-*
**PRSS — catastrophizing (0–6)**	MBCT Control	3.47 3.37	*(0.98)* *(1.09)*	3.11 3.31	*(0.93)* *(1.03)*	3.05 -	*(0.92)* *-*
**SCS (1–5)**	MBCT Control	3.09 2.87	*(0.65)* *(0.60)*	3.37 3.02	*(0.61)* *(0.72)*	3.29 -	*(0.80)* *-*
**FMI (14–56)**	MBCT Control	37.64 36.37	*(5.41)* *(4.95)*	39.77 35.66	*(4.68)* *(4.95)*	38.28 -	*(4.27)* *-*

*MBCT* intervention group, *Control* control group, *M* mean, *SD* standard deviation. *t*0 pre-intervention, *t*1 post-intervention, *t*2 7-month follow-up. Questionnaires: *PSQ* Perceived Stress Questionnaire, *HADS* Hospital Anxiety and Depression Scale, *PSRS* Perceived Stress Reactivity Scale, *DFS* Questionnaire of Dysfunctional and Functional Self-Consciousness, *PRSS* Pain-Related Self-Statements Scale, *SCS* Self-Compassion Scale, *FMI* Freiburg Mindfulness Inventory

Tables 3 and 5 display the results of the ANCOVAs, according to the headache and psychological variables. Table 4 displays the results of the linear mixed model for the Poisson distributed variables. The covariates included in the models were the three stratification variables (age, gender, self-reported number of migraine days per month) as well as the baseline scores of each variable.

**Table 3:** Results of group comparisons at *t*1 for the normally distributed headache-related variables including the primary outcome (univariate ANCOVA)

ANCOVA	MBCT *(n* = 24) *M*_adj_ *(SEM)*	Control (*n* = 24) *M*_adj_ *(SEM)*	*F*	*p*	Cohen’s *d*_adj_
**Impairment during a headache attack (0–10) *— Primary outcome***	4.03 (0.31)	4.41 (0.31)	0.734	.396	0.22
**Pain intensity during a headache attack (0–10)**	4.32 (0.26)	4.21 (0.26)	0.078	.782	−0.08

*M*_adj_ means at *t*1 adjusted for covariates (mean at baseline, stratification variables), *SEM* standard error of the mean, *F* test statistic for the ANCOVA, *p p*-value for the ANCOVA, Cohen’s *d*_adj_ effect size in terms of the standard deviation

**Table 4:** Results of linear mixed model of the Poisson distributed headache-related variables for *t*0 and *t*1

	**MBCT *(n* = 24) *M***_**adj**_ ***(SEM)***	**Control *(n* = 24) *M***_**adj**_ ***(SEM)***	***b***	***z-score***	***p***	**Cohen’s *d***_**adj**_
**Headache days per month**	6.07 (0.87)	8.27 (0.87)				0.38
*Group* × *time*			0.300	2.044	.041*	
*Group*			0.023	0.125	.90	
*Time*			−0.315	−2.893	.004**	
**Days with medication per month**	4.07 (0.46)	4.01 (0.46)				−0.03
*Group* × *time*			−0.169	−0.882	.378	
*Group*			0.228	1.380	.168	
*Time*			−0.136	−0.980	.327	

*M*_adj_ means at *t*1 adjusted for covariates (mean at baseline, stratification variables), *SEM* standard error of the mean, *b* unstandardized regression coefficient, *z*-score and respective *p*-value, Cohen’s *d*_adj_ effect size in terms of the standard deviation

*p* values: *p < .05, **p < .01

As shown in Table 3, the primary outcome of headache impairment reduction reveals a non-significant effect with a non-substantial effect size of *d*_adj_ = 0.22. Also, for the secondary outcome pain intensity, there was no significant difference between groups at the adjusted post-measurement. In Table 4 the frequency-based headache variables show a significant *time* × *group* interaction for the number of headache days (*p* = 0.04, *d*_adj_ = 0.38), indicating that the participants in the MBCT group had a significant reduction of headache days compared to controls. In contrast, the variable medication use showed no significant change (*p* = 0.38).

As shown in Table 5, the results of the psychological questionnaires are heterogeneous. Of the eight scales, four showed significant differences at post-measurement between groups. In the MBCT group, the participants reduced their level of perceived stress and anxiety significantly more than the participants in the control group (PSQ: *F*(1,48) = 6.084, *p* < 0.05; HADS-D-anxiety: *F*(1,48) = 4.891, *p* < 0.05). Effect sizes were falling in the small range with an adjusted Cohen’s *d* of 0.43 and 0.34, respectively. Moreover, MBCT participants reduced their amount of rumination and their amount of catastrophizing significantly more than the participants in the control group (DFS-rumination: *F*(1,48) = 7.75, *p* < 0.01; PRSS-catastrophizing: *F*(1,48) = 4.11, *p* < 0.05). Here, effect sizes were falling in the small to medium range with an adjusted Cohen’s *d* of 0.52 and 0.34, respectively.

**Table 5:** Results of the between-group comparisons at *t*1 for the psychological variables (ANCOVA)

	**MBCT *(n* = 27) *M***_**adj**_** (*SEM*)**	**Control *(n* = 27) *M***_**adj**_** (*SEM*)**	***F***	***p***	**Cohen’s *d***_**adj**_
**PSQ**	39.57 (2.24)	47.43 (2.24)	6.084	.017*	0.43
**HADS-D — anxiety**	5.99 (0.42)	7.33 (0.42)	4.891	.032*	0.34
**HADS-D — depression**	4.14 (0.41)	4.86 (0.41)	1.462	.233	0.24
**PSRS**	24.40 (0.92)	26.54 (0.92)	2.652	.11	0.26
**DFS — rumination**	2.86 (0.09)	3.22 (0.09)	7.750	.008**	0.52
**PRSS — catastrophizing**	3.04 (0.11)	3.37 (0.11)	4.107	.048*	0.34
**SCS**	3.29 (0.11)	3.10 (0.11)	1.443	.24	0.29
**FMI**	39.09 (0.97)	36.34 (0.97)	3.662	.062	0.49

*M*_adj_ means at *t*1 adjusted for covariates (mean at baseline, stratification variables), *SEM* standard error of the mean, *F* test statistic for the ANCOVA, *p p*-value for the ANCOVA, Cohen’s *d*_adj_ effect size in terms of the standard deviation Questionnaires: *PSQ* Perceived Stress Questionnaire, *HADS* Hospital Anxiety and Depression Scale, *PSRS* Perceived Stress Reactivity Scale, *DFS* Questionnaire of Dysfunctional and Functional Self-Consciousness, *PRSS* Pain-Related Self Statements Scale, *SCS* Self-Compassion Scale, *FMI* Freiburg Mindfulness Inventory

*p* values: *p < .05, **p < .01

### Follow-up

Due to the waitlist design only the intervention group had a follow-up measurement at 7 months. We computed repeated measurement analyses for all three timepoints. For variables with a significant time effect, a contrast analysis was conducted comparing *t*0 against *t*2. The full analyses of all variables and contrasts can be seen in Tables S1, S2 and S3 in the supplement file.

There was no significant effect for the primary outcome. Regarding secondary outcomes, we found a significant reduction in the number of headache and medication days. Contrasts from *t*0 to *t*2 were also significant (headache days *p* = 0.00002, *d* = 0.75, medication days *p* = 0.002, *d* = 0.46). Psychological functioning and levels of anxiety, depression, rumination, catastrophizing, and self-compassion showed significant improvement over time. Contrast analyses revealed significant effects for rumination (*p* = 0.02, *d* = 0.28), catastrophizing (*p* = 0.0005, *d* = 0.44), and self-compassion (*p* = 0.01, *d* = 0.27).

### Goal Attainment

The goals reported by the participants were aggregated to eight thematic clusters. It was shown that the most frequently named goal (29%, *n* = 10 people) was “learning relaxation and stress reduction.” The second most frequently named goal (20%, *n* = 7) was “body awareness in the sense of early recognition and regulation of symptoms.” These two goals were rated as being reached “as expected” (90%) or “much more than expected” (75%), demonstrating a relatively high degree of satisfaction in this regard. The third most frequently named goal (17%, *n* = 6) was “reduction in migraine intensity and frequency.” It was rated as being reached “as expected” or “much more than expected” by two people, while the majority of four people chose the category “less than expected”.

### Feasibility and Compliance

Seventeen out of 19 participants answered the questionnaire. The responses were as follows: 88% of participants refer to themselves as “rather satisfied” or “very satisfied” with the MBCT course. Ninety-four percent reported that they would *recommend the MBCT course to others* and that they would *continue to use the learned techniques* in their everyday life. Concerning *homework compliance for the formal techniques* such as sitting meditation or the body scan, 6% of the participants reported to have practiced every day, 35% several times a week, 41% several times a month, and 18% a few times on the whole. Concerning *homework compliance for the informal techniques*, 12% reported to have practiced daily, 47% several times a week, 23% several times a month, and 18% a few times on the whole. Asking participants about the *helpfulness of MBCT for dealing with their migraine*, 23.5% rated the learned techniques to be helpful during an acute attack. In contrast, 94% reported the techniques to be helpful for the prophylaxis of future attacks. Beyond the results of the questionnaire, dropout rates were studied to complete the feasibility assessment. Seven people (26%) withdrew from the course after starting it, and dissatisfaction with the course was the most frequent reason that was reported (*n* = 3; 11%). Adverse events due to course participation have not been reported by any of the patients in this trial.

## Discussion

Our RCT evaluated a migraine-specific MBCT program in a sample of moderately impaired migraine patients compared to a waitlist control group, assessing its influence on headache parameters and variables of psychological functioning and coping as well as its feasibility.

Our trial revealed mixed results. First of all, the MBCT program proved to be a feasible intervention in terms of personal contentment and compliance. Concerning the primary outcome of headache-related impairment, no group effect was found. Regarding secondary outcomes, several interesting effects could be found. With respect to the headache variables a significant reduction of headache days per month was found, while no effects were revealed for the other two headache variables. Concerning the variables of psychological functioning and coping, a superiority of the MBCT intervention was found in four out of eight outcomes with mainly small effects.

Overall, the results of our trial do not fit neatly into the existing literature on this topic. The fact that there were no results for the primary outcome of headache-related impairment is a notable contradiction to our own pilot study and to the RCTs of Day et al. [18], Wells et al. [25], and Seng et al. [24] (see “1”). These RCTs assessed mindfulness-based interventions (headache/migraine-adapted MBCT in two cases) concerning their effects on headache and migraine parameters and shared the conclusion that headache-related disability is affected by these interventions. Wells et al. and Seng et al. even proposed this outcome to be the major focal point or goal in mindfulness-based programs, with its implication of improving resilience even in the face of pain (see also [56]).

At this point, we have no firm explanation why this variable failed to produce similar results in our trial. One possible explanation might be the existence of a floor effect due to the assessment of *headache*-related and not *migraine*-related impairment. In the present trial impairment, also included are the days with low-threshold tension-type headaches that may be less impairing than migraine attacks. Accordingly, the baseline impairment rating was around four out of a maximum of 10, not leaving much space for improvement. Also, the use of acute medication may be an important moderating variable regarding impairment that cannot be compared to other studies due to lack of information. Our patients reported a high use of acute medication at baseline (96%). It can be concluded that — given the high efficacy of modern medication — the overall disability in a headache attack is substantially reduced by this factor.

Regarding the secondary outcomes one needs to consider that the trial was not powered to detect effects for these multiple outcomes. Consequently, findings in these variables need to be considered as exploratory and should be interpreted with caution. With respect to headache days, the participants in the MBCT program improved by more than 2 days compared to controls. Within the 7-month follow-up interval, they showed a reduction of 38%, or more than 3 headache days and a medium to large effect size compared to baseline. Gaining 3 headache-free days per month represents a considerable increase in quality of life. In addition, in pharmacological studies, a pain reduction of one-third to a half is usually seen as a clinically meaningful difference. Performing responder analyses, 54% of the MBCT participants showed a reduction of headache days of at least one-third, and 38% reported a reduction of at least a half between baseline and follow-up. This range of improvement in the within-group comparison is falling in the spectrum of the established behavioral-medical interventions for migraine of 32–55% [57–61].

The above-cited literature shows inconsistent results with regard to headache frequency, with the majority of studies revealing no significant effects. Our finding of a clinically meaningful decrease in headache frequency speaks in favor of the adapted MBCT being able to influence the migraine disease directly. As our MBCT program seems to have affected psychological functioning and coping skills as shown as part of our secondary outcomes, it can be suggested that this might in turn have reduced the emergence of headaches. This effect may be due to removing parts of the “breeding ground” or triggering potential for headaches and thus resulting in the significant headache reduction. Such hypotheses should be followed up with mediation analyses in future studies.

Concerning the psychological variables, in four out of eight outcomes a superiority of the MBCT intervention could be demonstrated. These four outcomes showed elevated baseline levels of stress, anxiety, and cognitive over-engagement in the form of rumination and catastrophizing, that are comparable to patient groups with, for example, psychosomatic complaints (PSQ), patients undergoing psychotherapy (DFS) or patients with lower back pain (PRSS). These findings suggest that the MBCT program, which is targeting tension regulation, cognitive defusion as well as stress and pain management [22, 62–64], addresses the special needs of people suffering from migraine.

Finally, the goal attainment assessment enabled us to draw deeper conclusions about the personal perceptions of our participants. The two most frequently named goals in the goal assessment rating were “learning relaxation and stress reduction” and “body awareness in the sense of early recognition and regulation of symptoms”. These goals were rated as either “achieved” or “achieved more than expected” by 90% and 75%, respectively. These results demonstrate what participants expected of the program and document the high degree of satisfaction with these goals. Nevertheless, for patients expecting a clear change of migraine intensity or impairment, the adapted MBCT program might not be appropriate.

Our study has several limitations. First of all, a waitlist control group does not allow for testing of the specificity of the program. It cannot be decided whether the observed changes are due to the more generic features of a regular group program or the specific mindfulness practices taught. Comparative effectiveness trials with interventions from the field of cognitive-behavioral treatments would be necessary to answer this question.

A second limitation is the sample size. The rate of participants who drop out from a study, sometimes even before the intervention starts, should be estimated higher in future studies. Although our sample size was based on the results of our pilot study, the study did not have sufficient power for the secondary variables.

A third limitation is the use of paper-and-pencil diaries. Electronic versions of diaries have shown a higher reliability compared to paper–pencil versions [65]. Generally, the sole use of a headache diary should be reconsidered. In addition to a daily Likert-scale rating system, other instruments like the MIDAS [66] could be used, which assess retrospectively the number of days per month in which a remarkable impairment occurs.

Another limitation is that our data analysis was not conducted in blinded manner, and this may have resulted in an allegiance bias. Finally, the patients learned about their group assignment during the baseline diary assessment. Unfortunately, it was not possible to assess a potential impact of the latter, as the precise date that each participant read the information sent by mail could not be tracked. However, a provisional split of the diaries into two halves revealed no differences. With respect to generalization, the results of our trial can only be seen in relation to migraine patients who share similar socio-demographic and clinical characteristics. These include a medium-level migraine frequency with still a good level of everyday functioning. They also include an attitude of openness and curiosity towards psychologically oriented interventions and a tolerance for high homework requirements.

Furthermore, our trial assessed migraine-related variables based on a headache diary since these measurements are more reliable than retrospective assessments based on questionnaires. However, the downside of this approach is that our findings cannot so easily be compared to trials applying retrospective measurements.

Finally, we should reflect on the methodology of evaluating interventions like MBCT on a meta-level. Being rooted in Buddhist traditions, mindfulness programs are not directed to (effectively) eliminating symptoms or pain, but they foster a more salutogenic encounter with and handling of pain and discomfort in the sense of “adding” more degrees of freedom to one’s experience and behavior patterns. To translate this more thoroughly into research methodology, symptom-oriented outcome variables might be even better complemented by variables aiming at salutogenic processes of coping and acceptance.

In conclusion, we found in our RCT no effect in the primary endpoint of headache-related impairment. On the other hand, the exploratory analyses of the secondary variables revealed that the participation in a migraine-adapted MBCT may be associated with substantial and prolonged reductions in headache frequency and other variables of psychological functioning and coping. Assessment of the patients’ perspectives revealed high levels of contentment and achievement of personal expectations related to course participation. From our trial data, we cannot recommend the adapted MBCT program to patients who primarily aim at reducing the impairment they experience from their headache. However, as the adapted MBCT program shows potential in influencing headache features and in fostering higher-level coping processes, it merits further evaluation in the field of headache disorders that cause suffering and impairment for millions of patients.

## Supplementary Information

Below is the link to the electronic supplementary material.

Supplementary file1 (DOCX 19 KB)
