# Supplementary material for: Mindfulness-Based Cognitive Therapy as Migraine Intervention: a Randomized Waitlist Controlled Trial
Source: Int J Behav Med. 2021 Dec 21;29(5):597–609. doi: 10.1007/s12529-021-10044-8 (PMC9525407; doi:10.1007/s12529-021-10044-8)
Supplement: Supplementary file 1 — Supplementary file1 (DOCX 19 KB) [file 12529_2021_10044_MOESM1_ESM.docx]

Mindfulness-Based Cognitive Therapy as Migraine Intervention –

A Randomized Controlled Trial

**Supplement**

**Within-group analyses and follow-up analyses for the intervention group**

The MBCT group was delivering three data sets at three measurement points, allowing comparisons over time on the within-group level. Repeated measures ANOVAs were conducted to test differences between baseline and post-measurement as well as between baseline and follow-up measurement. In case of violation of the sphericity assumption in repeated measures analyses, the Greenhouse-Geisser-correction was applied. If an overall effect occurred, contrast analyses were conducted with the option of simple contrasts testing the pre-scores against the post- and follow-up scores. In case of frequency data the change over the three time points was assessed with the use of the linear mixed model. Cohen´s *d* was chosen as effect size measure.

Table S1: Results of the within-group comparisons (MBCT group) for the headache-related variables (repeated measures ANOVA)

| **Repeated measures ANOVA (MBCT: *n* = 24)** | | |
| --- | --- | --- |
|  | **Omnibus test** | |
|  | *F_omnibus_* | *p_omnibus_* |
| **Impairment during a headache attack (0-10)** | 0.467 | .63 |
| **Pain intensity during a headache attack (0-10)** | 1.121 | .34 |

*F*_omnibus_: test statistic for the ANOVA omnibus test; *p*_omnibus_: *p*-value for the ANOVA omnibus test

Table S2: Results of the within-group comparisons (MBCT group) for the headache-related variables (linear mixed model including contrasts)

| **LMM (MBCT: *n* = 24)** | | | | | | |
| --- | --- | --- | --- | --- | --- | --- |
|  | **Omnibus test** | | **contrasts**  **t0 – t1** | | **contrasts**  **t0 – t2** | |
|  | *Χ²* | *p* | *p* | *d* | *p* | *d* |
| **Number of headache days per month** | 19.394 | .00006*** | .004** | 0.53 | .00002*** | 0.75 |
| **Number of days with medication per month** | 3.833 | .15 | .104 | 0.23 | .002** | 0.46 |

*p* values: ** = *p* < .01, *** = *p* < .001; *Χ²*: test statistic for the GLMM omnibus test; *p*: *p*-value for the GLMM omnibus test /contrasts; *d*: effect size in terms of the standard deviation (Cohen´s *d*).
*t0*: pre-intervention; *t1*: post-intervention; *t2*: 7-month follow-up

Table S3: Results of the within-group comparisons for the psychological variables (repeated measures ANOVA including simple contrasts)

| **Repeated measures ANOVA (MBCT: *n* = 27)** | | | | | | | | | |
| --- | --- | --- | --- | --- | --- | --- | --- | --- | --- |
|  | **Omnibus test** | | **Simple contrasts**  **t0 – t1** | | | | **Simple contrasts**  **t0 – t2** | | |
|  | *F_omnibus_* | *p_omnibus_* | *F* | *p* | *d* | *F* | | *p* | *d* |
| **PSQ** | 3.000 | .06^T^ | 5.450 | 0.014* | 0.37 | 0.680 | | 0.209 | 0.13 |
| **HADS-D - Anxiety** | 4.088 | .03* | 1.352 | 0.128 | 0.15 | 1.966 | | 0.087 | -0.19 |
| **HADS-D - Depression** | 3.659 | .03* | 1.123 | 0.15 | 0.15 | 2.460 | | 0.065 | -0.23 |
| **PSRS** | 2.474 | .09^T^ | 4.957 | .02* | 0.28 | 0.946 | | .17 | 0.14 |
| **DFS - Rumination** | 4.360 | .02* | 6.664 | .008** | 0.41 | 5.328 | | .02* | 0.28 |
| **PRSS - Catastrophizing** | 6.425 | .003** | 8.094 | .005** | 0.38 | 14.894 | | .0005*** | 0.44 |
| **SCS** | 5.006 | .01* | 7.498 | .006** | 0.44 | 5.708 | | .01* | 0.27 |
| **FMI** | 3.010 | .06 ^T^ | 5.308 | .02* | 0.42 | 0.477 | | .25 | 0.13 |

*p* values: T= p < .10, * = *p* < .05, ** = *p* < .01, *** = *p* < .001

*F*: test statistic for the ANOVA omnibus test / contrasts; *p*: *p*-value for the ANOVA omnibus test / contrasts; *d*: effect size in terms of the standard deviation (Cohen´s *d*).

*t0*: pre-intervention; *t1*: post-intervention; *t2*: 7-month follow-up.

Questionnaires: PSQ: Perceived Stress Questionnaire, HADS: Hospital Anxiety and Depression Scale, PSRS: Perceived Stress Reactivity Scale, DFS: Questionnaire of Dysfunctional and Functional Self-Consciousness, PRSS: Pain-Related Self Statements Scale, SCS: Self-Compassion Scale; FMI: Freiburg Mindfulness Inventory
